# Exploring the gelling properties of Plantago ovata-based Arabinoxylan: Fabrication and optimization of a topical emulgel using response surface methodology

**DOI:** 10.1371/journal.pone.0290223

**Published:** 2023-08-22

**Authors:** Arshad Mahmood, Alia Erum, Ume Ruqia Tulain, Nadia Shamshad Malik, Aneeqa Saleem, Mohammed S. Alqahtani, Muhammad Zubair Malik, Mahwish Siddiqui, Asif Safdar, Abdul Malik

**Affiliations:** 1 College of Pharmacy, Al Ain University, Abu Dhabi, UAE; 2 AAU Health and Biomedical Research Center (HBRC) Al Ain University, Abu Dhabi, UAE; 3 Faculty of Pharmacy, University of Sargodha, Sargodha, Pakistan; 4 Faculty of Pharmacy, Capital University of Science and Technology, Islamabad, Pakistan; 5 Department of Pharmaceutics, Nanobiotechnology Unit, College of Pharmacy, King Saud University, Riyadh, Saudi Arabia; Siksha O Anusandhan University School of Pharmaceutical Sciences, INDIA

## Abstract

Prime objective of the current research was to develop a stable nimesulide emulgel with the help of arabinoxylan, a natural gelling agent extracted from Plantago ovata. The response surface methodology was used by a Design Expert 10 software to formulate and optimize the emulgel. The experimental design approach evaluated the impact of independent and dependent variables. Independent variables were different concentrations of arabinoxylan, span 80 and tween 20, whereas, dependent variables were viscosity, pH, and content uniformity. FTIR demonstrated the compatibility of nimesulide with the excipients. Stability study indicated no phase separation and no change in pH for formulation F1, F3 and F4. The negative values of zeta potential revealed the excellent stability of emulgel. Viscosity, spreadability and extrudability values were in desired range. Ex-vivo permeation study illustrated 86%, 55% and 66% release of the drug over a period of 24 h from the formulations F1, F3 and F4, respectively. Analgesic effect of the optimized emulgel was significantly higher in test group as compared to control and did not produce any sort of irritation. Therefore, it can be concluded that the newly developed emulgel based on arabinoxylan, as gelling agent, appear to be an effective drug delivery system.

## 1. Introduction

Human skin, being one of the largest and unique organs in the human body, covers approximately 10% of the body mass and has an average area of 1.7 m^2^ [1]. This organ presents an excellent opportunity for the administration of therapeutic agents, both for local and systemic effects. However, orally administered drugs for skin diseases often exhibit poor bioavailability and are associated with undesirable side effects. To address these limitations, topical drug delivery systems have gained significant interest [2]. These systems offer several advantages over oral delivery, as they allow direct application of drugs to the affected area, avoiding first-pass metabolism and minimizing systemic exposure. Among the various topical drug delivery systems, gels have emerged as one of the most popular semisolid formulations in recent times [3].

Gels are a type of dosage form that primarily consists of aqueous components, enabling easy drug release through aqueous vehicles [4]. However, one of the major limitations of gels is their inability to effectively deliver hydrophobic drugs, as they are primarily designed for the entrapment and delivery of hydrophilic drugs [5]. This poses a challenge when it comes to incorporating hydrophobic or lipophilic drugs into gel systems [6]. To address this limitation and enable the delivery of hydrophobic drugs, a modified formulation called "emulgel" has been developed and gained attention [7]. Emulgel combine the advantages of both emulsions and gels, allowing for the incorporation of hydrophobic drugs within a gel matrix. Emulgel formulations consist of a dispersed phase containing the hydrophobic drug, typically in the form of oil droplets, dispersed within the gel structure. The presence of emulsifying agents helps in stabilizing the emulsion and preventing phase separation.

Emulgel is an innovative and promising topical drug delivery system that offers various advantages over other conventional topical formulations. It is characterized by its greaseless nature, easy spreadability, and soothing properties, making it safe, biocompatible, and visually appealing. Compared to other topical drug delivery systems, emulgel provide a superior and effective means of delivering drugs. The formulation of emulgel involves the combination of an oil phase, gelling agent, aqueous phase, and emulsifying agent, which significantly influences the drug release profile from the dosage form [8]. The unique aspect of emulgel is their dual character, encompassing both an emulsion and a gel base within a single formulation. This distinctive feature enables emulgel to efficiently deliver drugs with diverse solubility profiles, including both hydrophilic and lipophilic drugs [9]. One of the key advantages of emulgel is their ability to control the drug release pattern by manipulating the emulsion base. This allows for the sustained and prolonged therapeutic effect at the targeted site of action. The drug, when delivered through an emulgel, is brought in close proximity to the skin surface, resulting in a higher concentration gradient and enhanced skin penetration. The emulgel formulation exhibits a high solubilizing capacity, facilitating improved drug permeation through the skin [10].

In recent times, emulgel have gained considerable attention for the delivery of a wide range of drugs with diverse pharmacological effects. They have been successfully utilized for delivering hydrophilic and hydrophobic drugs, including non-steroidal anti-inflammatory drugs, anti-emetics, anti-fungal agents, antiviral drugs, antibacterial, and local anesthetics [11]. The versatility of emulgel allows for the incorporation of various pharmacologically active substances, expanding their potential applications in different therapeutic areas.

Natural polymers and their derivatives have gained significant attention in drug delivery research due to their biodegradability, cost-effectiveness, and potential for chemical modification [12–14]. These advantages make them attractive alternatives to synthetic polymers. Among these natural polymers, arabinoxylan (AX) stands out as a particularly promising candidate. AX can form highly viscous solutions and gels at low concentrations (0.5–1.5%), which not only contribute to its excellent gelling properties but also make it cost-effective compared to commercially used gelling agents. The availability of AX from renewable sources further enhances its cost-effectiveness and sustainability. Moreover, AX exhibits thixotropic behavior, meaning it can recover its gel structure after the applied stress is removed, offering additional advantages in terms of ease of application and stability. Overall, the cost-effectiveness of AX, combined with its unique rheological properties, make it a valuable natural polymer for a wide range of drug delivery applications. [15–17].

Our research work focused on utilizing the gelling properties of arabinoxylan (AX) to develop an efficient emulgel for topical drug delivery. We incorporated nimesulide, a model drug effective for pain associated with osteoarthritis and backache, into the emulgel formulation. Through comprehensive evaluations, including pH, viscosity, spreadability, swelling index, content uniformity, ex-vivo permeation study, as well as in vivo skin irritation and analgesic activity, we investigated the performance and potential therapeutic applications of the nimesulide emulgel. The developed emulgel formulation combines the advantages of both an emulsion and a gel, offering improved drug solubility, enhanced drug permeation, and extended residence time on the skin. This emulgel formulation holds promise for topical application, delivering targeted relief from pain and inflammation to the affected area. Hence, present study provides unique insights into the formulation development, optimization, and performance evaluation of the emulgel containing nimesulide.

The inclusion of two emulsifying agents, Span 80 and Tween 20, in our emulgel formulation is based on the rationale of harnessing their synergistic effects to achieve stable emulsions and desirable rheological properties. Span 80, a lipophilic emulsifier, stabilizes oil-in-water emulsions and ensures uniform distribution of the oil phase. Tween 20, a hydrophilic emulsifier, promotes oil-in-water emulsion formation and provides a uniform dispersion of the water phase. By combining these emulsifiers, we optimize the emulsification process, enhance emulsion stability and improve product appearance. The use of both emulsifying agents contributes to the overall effectiveness and stability of our emulgel formulation.

## 2. Materials and methods

Nimesulide was received as a gift sample from Unixo Labs pharmaceuticals. AX was extracted from (*Plantago ovata*) that was purchased from the local market of Sargodha (Sargodha, Pakistan). Acetic acid, span80, tween 20, and sodium dihydrogen phosphate and sodium hydroxide were purchased from Sigma Aldrich, Germany. Propylene glycol was purchased from Anala R. England. Ethanol was purchased from Riedel-de Haen, Germany. Liquid paraffin was purchased from Merck Millipore and methylparaben was purchased from Sigma Aldrich, USA. Distilled water was taken from the Industrial plant of the University of Sargodha (Sargodha, Pakistan). All other chemicals used in this study were of analytical grade.

### 2.1. Extraction of AX from Plantago ovata

AX was extracted from Plantago ovata seeds using the alkali extraction method as previously described by Saghir *et al*. [17]. In this method, 150g of Plantago ovata seeds were soaked in 500 mL of distilled water overnight. The pH of the solution was then adjusted to 12 by adding 2.5% aqueous NaOH and stirred for 5 minutes. The mixture was separated using vacuum filtration, and the filtrate was treated with dilute acetic acid until the pH reached a range of 3–4 to induce coagulation. The resulting gel was washed with distilled water for 4 days and subsequently freeze-dried to obtain the AX extract.

### 2.2. Experimental design of emulgel formulations using response surface methodology

As illustrtaed in Table 1, fifteen formulations (F1 to F15) were designed based upon different concentrations of AX, span 80 and tween 20, being considered the independent variables and their concentrations were chosen by a review of the literature [18–20]. The selection of the ratio factors for AX, span 80 and tween 20 were decided using, Design Expert 10, software, Response surface methodology (Stat-Ease, Inc., Minneapolis, Minnesota, USA) to get optimize emulgel formulations.

**Table 1:** Formulation design of emulgel and dependent variables (Viscosity, pH and drug content).

Formulation code	Arabinoxylane (%w/w)	Span 80 (%w/w)	Tween 20 (%w/w)	Viscosity (Cps) mean + SEM	pH mean + SEM	Drug Content (%) mean + SEM
F1	1.5	1.5	0.75	17987 ± 2.08	5.6 ± 0.03	106 ± 0.06
F2	1.5	2	0.75	16548 ± 49.9	5.9 ±0.03	98 ± 0.31
F3	2	2	0.5	29857 ± 504.79	6.8 ± 0.08	115 ± 0.05
F4	1	1	1	11445 ± 70.40	6.1 ± 0.03	85 ± 0.28
F5	1.5	1.5	0.5	18377 ± 35.37	5.8 ± 0.05	105 ± 0.52
F6	2	1	1	28765 ± 127.69	6.5 ± 0.08	92 ± 0.57
F7	1	1.5	0.75	13654 ± 128.77	6.4 ± 0.03	60 ± 0.88
F8	2	2	1	30676 ± 89.29	6.4 ± 0.03	104 ± 0.80
F9	1	1	0.5	12543 ± 15.16	6.4 ± 0.04	85 ± 0.30
F10	2	1	0.5	26378 ± 90.35	6.1 ± 0.03	89 ± 0.90
F11	1.5	1	0.75	16424 ±45.33	6.3 ± 0.03	97 ± 0.30
F12	1	2	1	12543 ± 154.70	6.6 ± 0.18	90 ± 0.0
F13	1.5	1.5	1	16980 ±80.55	6.1 ± 0.03	103 ± 0.6
F14	2	1.5	0.75	28543 ± 273.20	6.6 ± 0.04	68 ± 0.31
F15	1	2	0.5	13658 ± 22.66	6.4 ± 0.03	92 ± 0.31

The concentration of independent variables AX, span 80 and tween 20 were denoted by A, B and C, respectively. The points for independent variables comprised of lower (-1), medium (0), and higher (+1). For AX and span 80, concentration -1 was assigned to designate minor level concentration of 1%, for medium-level 0 was used for 1.5% and maximum concentration +1 was used for 2.0%. Similarly, for the concentration of tween 20, -1 indicates a lower concentration 0.5%, 0 indicates a medium concentration 0.75% and +1 indicates a higher concentration of 1%. Intercept is indicated as follows;

$$\text{A}+\text{B}+\text{C}+\text{AB}+\text{AC}+\text{BC}+{\text{A}}^{2}+{\text{B}}^{2}+{\text{C}}^{2}.$$

### 2.3. Preparation of Emulgel

From the formulation perspective, polymeric gel base dispersion was initially prepared by dispersing AX in distilled water with constant stirring at 510–550 RPM until the gel was formed and the pH was maintained at 6.5 ± 0.2 using 2.5% aqueous NaOH. For the emulsion, oil phase was prepared by dissolving span 80 in liquid paraffin and aqueous phase by dissolving tween 20 in distilled water. Separately, nimesulide was dissolved in ethanol and methylparaben in propylene glycol owed to the solubility of both ingredients in respective solvent. Both solutions of nimesulide and methylparaben were added to the aqueous phase. In the following, the oily and aqueous phases were separately heated in a water bath at 70°C. Upon obtaining uniformity, drug loaded aqueous phase was added to the oil phase with continuous stirring until the temperature reached to room temperature. The drug-loaded emulsion was incorporated into the polymeric gel base dispersion of AX steadily with continuous stirring to obtain homogenized emulgel. [19,20]. Afterwards, the three dependent variables (responses) viscosity, pH and drug assay were monitored and recorded.

### 2.4. Checkpoint analysis and optimization model validation

Design Expert 10, software (Stat-Ease, Inc., Minneapolis, Minnesota, USA), completed statistical validation of the polynomial equations, which was determined using the software’s ANOVA requirement. The models were assessed using R^2^ values and statistically significant coefficients. The optimal formulations were chosen based on their attractiveness as determined by Design-Expert software acceptance criteria. The Design-Expert software featured a variety of 3D reaction surface graphs. To validate the experimental model and polynomial equations, three optimal checkpoint formulations were chosen over the experimental region. The response qualities of the improved checkpoint formulation variables were tested. To compute the percentage prediction error, the experimental values of the answers were quantitatively compared to the projected values.

### 2.5. Characterization of emulgel

#### 2.5.1. Physical appearance

Visual inspection of formulations was done for their homogeneity, appearance, color, phase separation, and consistency [21].

#### 2.5.2. Viscosity study and pH determination

A viscosity test of all formulations was performed on a Brookfield viscometer (Brookfield, Massachusetts, USA) at 50 rpm and 100 rpm over a period of 10 min. The pH of all formulations was measured by using a digital pH meter [22].

#### 2.5.3. Drug content measurement

The amount of drug entrapped into the emulgel formulations was determined photometrically. Briefly, 1 g of each formulation was mixed with phosphate buffer (pH 7.4) (1:100 w/v) in a volumetric flask and continued stirring for 30 min. The mixture was further diluted with phosphate buffer (1:10 v/v) before measuring the absorbance using a UV spectrophotometer (Shimadzu, UV-2401 PC, Japan) against 392nm. A standard curve of the pure drug within the range of 1–10 μg/mL in phosphate buffer was used for quantification.

#### 2.5.4. Stability study

The stability studies were performed for all the formulations stored in well-closed glass containers for a period of 90 days at room temperature. Samples were collected at predetermined intervals 0, 30, 60 and 90 days, and their physicochemical parameters were evaluated [23].

#### 2.5.5. Spreadability measurement

A spreadability test based on ‘Slip’ and ‘drag’ characteristics of emulgel was performed for the optimized formulations. For this purpose, a glass slide was fixed on the wooden box and 1 g of each formulation was placed on this slide, separately. A second glass slide was used to cover in such a way that the emulgel was sandwiched between two glass slides. Afterwards, a standard weight of 1 Kg was placed on the sandwiched slides in order to remove any entrapped air and to get a uniform layer emulgel. After an incubation of 5 min, the excess emulgel that drained out of the slides was scrapped off and the weight was uplifted. In the following, the time required for the separation of two slides was noted in seconds [24].

The following formula was used to calculate spreadability,

$$S=\frac{M\times L}{T}$$ (1)

Whereas; S = Spreadability, M = Weight Knotted to slide, L = Length of glass slide, T = Time required for the separation of two slides.

#### 2.5.6. Extrudability measurement

As a measure of thickness of the emulgel formulations, extrusion from lacquered aluminum collapsible tube on application of weight (in g) required to extrude at least 0.5 cm ribbon of emulgel in 10 s was measured. The measurement of extrudability (g/cm^2^) of each formulation was done in triplicates [25].


$$Extrudability=\frac{Applied\;weight\;to\;extrude\;emulgel\;from\;tube\;(g)}{Area\;in\;cm2}$$ (2)

#### 2.5.7. Particle size (PS), polydispersity index (PDI), and zeta potential (ZP) determinations

A Malvern zeta-sizer (Worcestershire, UK) was used to determine the average globule size, polydispersity index, and zeta potentials of optimized emulgel formulations as per the concepts of dynamic light scattering and electrophoretic mobility theories, respectively.

#### 2.5.8. Photomicrography

Under a light microscope, the morphology of the emulsion was examined. A formulation that displayed the best release was examined for shape, globule size, and agglomeration. The formulation was mounted on a glass slide and viewed under a microscope.

#### 2.5.9. Fourier-Transform Infrared Spectroscopy (FTIR)

FTIR of pure Nimesulide, AX, and physical mixture of AX and nimesulide was done by KBr disc method by employing Bruker FTIR (Tensor 27 series, Bruker Corporation, Germany) instrument, using attenuated total reflectance (ATR) technology accompanying software OPUS data collection. All the spectrum was obtained at 400-4000cm^−1^ under similar operating conditions [26].

#### 2.5.10. Ex-vivo permeation study

The ex-vivo permeation study for the emulgel formulation was performed using a Franz diffusion cell (PermeGear, Inc. 1815 Leithsville Road, Hellertown, PA 18055 USA), following established protocols described in the literature (28–30).

Rabbit skin required for present study was obtained from the local animal house. The hair at the dorsal part of the animal was shaved carefully using an electric clipper followed by scarifying the rabbits and separating the skin. The adipose tissue was removed from the skin then it was preserved at 4°C in phosphate buffer maintained at pH 7.4. For experiment, rabbit skin was thoroughly rinsed with normal saline and then clamped between the donor and receptor chambers of the Franz cell.

A predetermined amount of the emulgel formulation (1 gram) was applied evenly over the rabbit skin. Phosphate buffer solution (pH 7.4) was used as the diffusion medium and maintained at a temperature of 37°C±0.5°C throughout the experiment. The diffusion medium was continuously stirred using a magnetic stirrer to ensure uniform distribution. Samples (0.1 mL) were collected at specific time intervals (30 minutes, 1 hour, 2 hours, 4 hours, 6 hours, 12 hours, and 24 hours), and an equal volume of pre-warmed buffer was immediately replaced after each sampling to maintain sink conditions. The collected samples were appropriately diluted and their absorbance was measured using a UV spectrophotometer. The cumulative amount of drug permeation through the skin was calculated as a function of time, allowing for the evaluation of the emulgel formulation’s permeation characteristics [27].

#### 2.5.11. In-vivo study

All the protocols for the in-vivo study were pre-approved by the Ethical Committee of the Faculty of Pharmacy, University of Sargodha (PREC4532). The in vivo experiment for analgesic activity was conducted on healthy adult Wistar rats weighing 150–200 g, whereas skin irritation was conducted on healthy albino rabbits weighing about 1.5 ± 0.5 kg. Animals were allowed to acclimatize for one week before the experiment. Animals were kept at ambient conditions (room temperature of 25 ± 2°C, relative humidity of 65 ± 5%, and light/dark cycle of 12 h) and had free access to standard pellet diet and fresh water [28]. Guidelines for care and use of laboratory animals of Faculty of Pharmacy, University of Sargodha, Sargodha, Pakistan, were used to perform animal study. Humane endpoints were established to safeguard animal welfare, ensuring that animals experiencing predetermined levels of distress were promptly removed from the study to minimize unnecessary suffering.

*2*.*5*.*11*.*1*. *Analgesic activity*. Two groups of rats were made, three rats per group. Group 1 was considered as standard group and group 2 was considered as test group. Group 1 or the standard group was treated with the marketed formulation of nimesulide. Group 2 or the test group was treated with the topical formulation of emulgel. *In-vivo* analgesic activity of nimesulide was examined by the Hot plate method. Each rat was kept on a hot plate (55°C) for 10 sec and the latency period (period at which animals show the reaction to the pain stimulus) or PRT (pain reaction time) was measured. 0.5 g of the respective formulation was applied on the dorsal surface of the right hind paw of rat with gentle rubbing. The time (in seconds), spent licking the paw was considered an indicator of pain response.

*2*.*5*.*11*.*2 Skin irritation test*. The test was conducted to explore the safety and skin compatibility of the optimized formulation. Two groups of rabbits were made, three rabbits each group. Group I was considered as control group, received no treatment while group II was considered as test group, and received topical treatment of emulgel.

Before the study, rabbits were shaved and circumscribed dorsal skin area. A sample (0.5 g) of the formulation was applied once daily for 7 days, and the rabbit’s skin was inspected for any signs of irritation, redness, and inflammation. A scoring system was considered and average scores were recorded. The scoring system was designed as follows; 0: no signs of irritation, 1: slight irritation, 2: moderate irritation, and 3: severe irritation [29].

#### 2.5.12. Statistical analysis for *In-vivo* study

For *In-vivo* study, the statistical analysis of all the result was accomplished utilizing GraphPad Prism 7.0 Graph Pad Software (San Diego, CA, USA). The data were analyzed using student t test. P value of <0.05 was considered as significant. All the results are depicted as value Mean± SEM.

## 3. Results and discussion

### 3.1. Formulation and optimization design by RSM

The experimental design was used for the optimization of the formulation variables, as the response surface methodology required 18 experiments. As illustrated in Table 2, dependent (A, B and C indicating Arabinoxylan, Span 80 and Tween 20 respectively also depicted their minimum, medium and maximum levels) and independent variables (R1,R2 and R3 indicating viscosity, pH & drug contents respectively). Table 3 indicates co-efficient for responses (a second-order polynomial equation defines the experimental link between independent variables and their responses in comparison to actual factors) and Table 4 comprises numerical optimization of formulations.

**Table 2:** Selection of ranges for dependent and independent variables.

**Dependent**								
**Factor**	**Name**	**Min**	**Max**	**Coded Low**		**Coded High**	**Mean**	**Std. Dev.**
A	IAX	1.0000	2.00	-1 ↔ 1.00		+1 ↔ 2.00	1.50	0.3835
B	Span 80	1.0000	2.00	-1 ↔ 1.00		+1 ↔ 2.00	1.50	0.3835
C	Tween 20	0.5000	1.0000	-1 ↔ 0.50		+1 ↔ 1.00	0.7500	0.1917
**Independent**								
**Response**	**Name**	**Units**	**Analysis**	**Min**	**Max**	**Mean**	**Std. Dev.**	**Model**
R1	Viscosity	cps	Polynomial	11445	30676	19440.00	6518.31	Quadratic
R2	pH	pH	Polynomial	5.6	6.8	6.24	0.3071	Quadratic
R3	drug assay	%	Polynomial	60	115	94.22	12.41	Quadratic

**Table 3:** Co-efficient Table for responses.

	Intercept	A	B	C	AB	AC	BC	A^2^	B^2^	C^2^
**Viscosity**	17957.3	8038	772.3	-40	397.625	676.875	-197.625	3567.11	-1045.39	147.107
**p-values**		< 0.0001	0.1601	0.938	0.496	0.2594	0.7322	0.0058	0.307	0.8818
**pH**	6.01071	0.05	0.07	0.02	0.0125	0.0125	-0.0375	0.453571	0.0535714	0.0964286
**p-values**		0.5978	0.464	0.8316	0.9053	0.9053	0.7221	0.0319	0.7672	0.5964
**drug assay**	95.9881	6.6	5.1	-1.2	3.25	-0.75	-2	-22.2262	6.27381	12.7738
**p-values**		0.0152	0.0447	0.5913	0.2125	0.7625	0.4286	0.0007	0.1665	0.0147

**Table 4:** Numerical optimization of formulations.

Name	Goal	Lower	Upper limit	Lower	Upper	Importance
		limit		weight	weight	
AX	Maximum	1	2	1	1	3
Span 80	Within range	1	2	1	1	3
Tween 20	Within range	0.5	1	1	1	3
Viscosity	Within range	11445	30676	1	1	3
pH	Within range	5.6	6.8	1	1	3
Drug assay	Maximum	60	115	1	1	3

Optimal conditions for three dependent variables (responses) were predicted by the desirability function and appropriate concentration ranges were chosen. Five options that are used by the program to shape the desirability indices include none, minimum, maximum, within range, and target. Our focus was to make formulations with good viscosity, acceptable pH range, and content uniformity. According to established criteria, the optimum situations for our formulation’s desired response were; the concentration of AX at 1.5%, the concentration of span 80 was 2% and the concentration of tween 20 was 0.75%. Design-Expert software was used to obtain optimized formulations and create the mathematical equations shown in (3), (4), and (5). The responses, viscosity (*Y*1) and pH (*Y*2), and percent drug diffusion (Y3) were found to be significantly higher (*Y*1, 30676) in F8; (*Y*2 6.8) and (Y3 115%) in F3 respectively. The response surface models were developed with Design-Expert software by applying coded values of factor levels to estimate the quantitative impacts of various combinations of variables on Viscosity, pH, and percent drug diffusion. The following is a representation of the model described:

$$\text{Viscosity}=17957+8038\text{AX}+772.3\text{span-}40\text{tween}20+397.625\text{AX}\times \text{span}+676.87\text{AX}\times \text{tween-}197.62\text{span}\times \text{tween}+3567.11{\text{AX}}^{2}-1045.39{\text{span}}^{2}80+147.107{\text{tween}}^{2}$$ (3)

$$\text{pH}=6.01+0.05\text{AX}+0.07\text{span}+0.02\text{tween}+0.0125\text{AX}\times \text{span}+0.0125\text{AX}\times \text{tween-}0.037\text{span}\times \text{tween}+0.453{\text{AX}}^{2}+0.053{\text{span}}^{2}-0.096{\text{tween}}^{2}$$ (4)

$$\text{DrugAssay}=95.98+6.6\text{AX}+5.1\text{span-}1.2\text{tween}+3.25\text{AX}\times \text{span-}0.75\text{AX}\times \text{tween-}2\text{span}\times \text{tween-}22.22{\text{AX}}^{2}+6.27{\text{span}}^{2}+12.77{\text{tween}}^{2}$$ (5)

### 3.2. Fitting of data to the model

**Fig 1:**
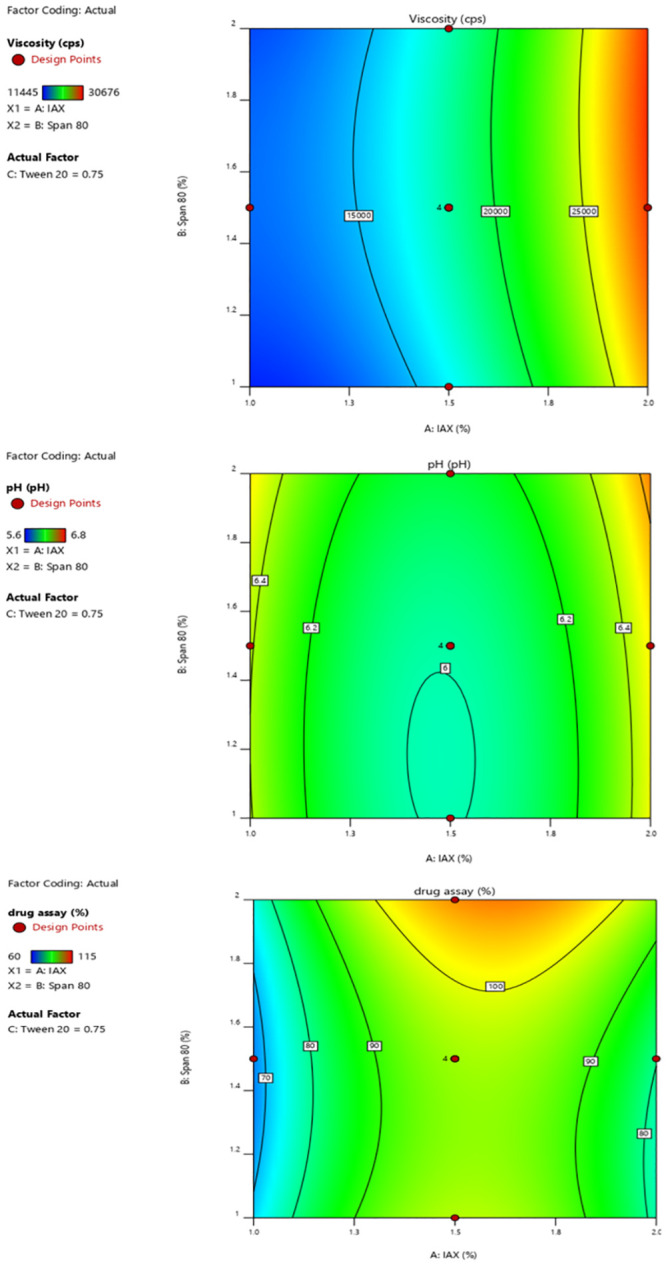
Contour plot of viscosity, pH and drug assay.

**Fig 2:**
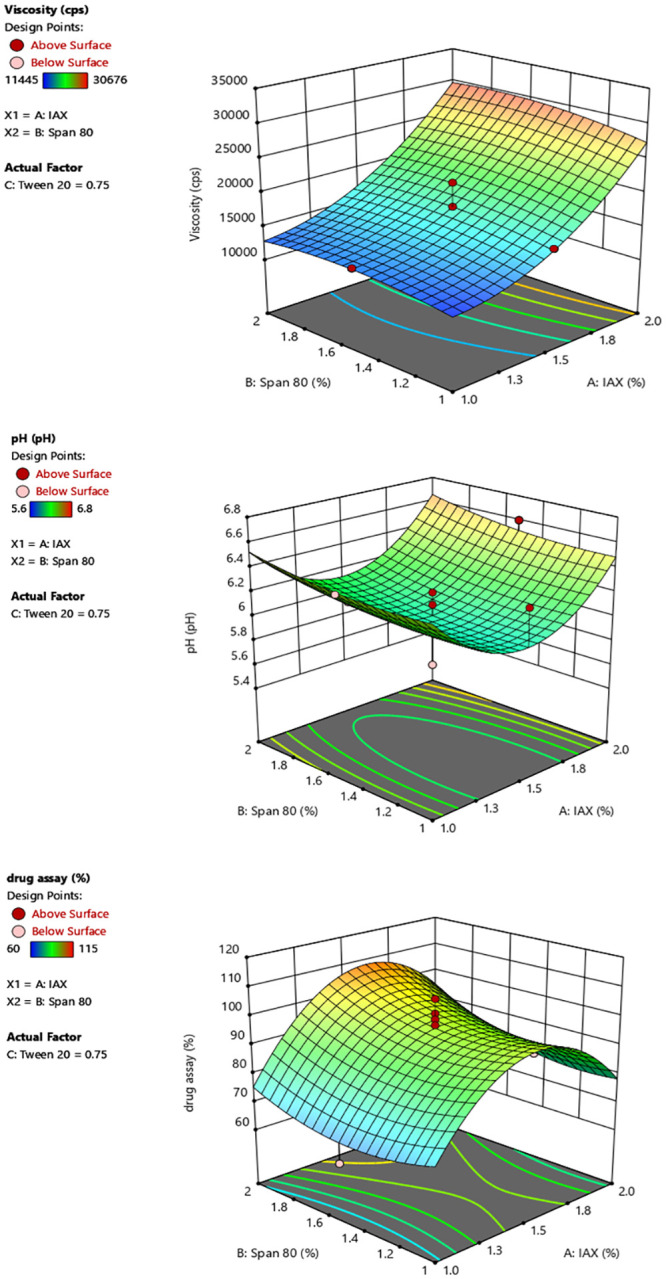
3-D graph of viscosity and drug assay.

Formulation F2 showed a significant percent drug diffusion (*Y*3) and pH (*Y*2) and Viscosity (Y1) among the formulations and demonstrated good quality with the best spreadability. Using Design Expert 10, software (Stat-Ease, Inc., Minneapolis, Minnesota, USA), the responses observed for 18 different formulations were fit to a design model at the same time as in Fig 1. A negative number implies that the effect favors the optimization, whereas a positive value suggests that the factor and the response have an inverse connection. The independent variable (gelling agent concentration) was discovered to have a negative influence on the responses: percent drug diffusion (Y3) as well as spreadability. To assess the impact of the independent factors on response and choose the best formulation, three-dimensional response surface plots were created as described in Fig 2.

### 3.3. Data analysis

Multiple regression was used to generate a polynomial equation using the percentage drug diffusion and viscosity acquired at various values of the three independent variables (X1, X2, and X3), the results are displayed in Table 5. The correlation coefficient (R^2^) of viscosity and pH was determined to be 0.9535 and 0.9273 respectively, suggesting that the selected variables for each are well matched. Moreover, the "Pred R-Squared" values fairly agree with the "Adj R-Squared" for both of them. The percentage of drug diffusion values tested for various formulations exhibited a broad range of values (i.e. from a minimum of 60.8% to a maximum of 115.5%), and the R^2^ was determined to be 0.7951. The "Pred R-Squared" is 0.6459, which is reasonably close to the "Adj R-Squared" of 0.7010. In general, the findings demonstrate that the factors chosen for the investigation have a significant impact on the percentage drug diffusion and when two variables are adjusted at the same time, the interaction terms reveal how the percentage drug diffusion varies. Positive coefficients for interactions between two variables indicate an adverse influence on percent drug diffusion, whereas negative coefficients for all three independent variables suggest a beneficial effect on percent drug diffusion.

**Table 5:** Summary of results of regression analysis for responses 1, 2 and 3.

Model	R^2^	Adjusted R^2^	Predicted R^2^	SD
Viscosity (*Y*1)	0.9535	0.9415	0.9160	0.43
pH (*Y*2)	0.9273	0.9193	0.8351	0.87
Drug content (%) (*Y*3)	0.7951	0.7010	0.6459	1.06

### 3.4. Percentage yield of arabinoxylan from Plantago ovata

AX was obtained from Plantago ovata using the alkali extraction method, resulting in a yield of 39%. Mild alkali treatment resulted in a strong gel formation, while harsh alkali treatment led to decreased viscosity and elasticity of the gel. This decrease in gel properties was attributed to the reduced ferulic acid (FA) content in AX caused by harsh alkali treatment. The FA content in AX plays a crucial role in determining the gel structure, with higher FA content leading to smaller mesh sizes, lower molecular weight, and increased cross-linking capacity, resulting in more compact gel structures. [30–32] The molecular weight of arabinoxylan (AX) obtained using the alkali extraction method was described by Saghir *et al*. The molar mass of AX was determined using Gel Permeation Chromatography (GPC), which is a technique commonly employed to analyze the size and distribution of macromolecules. The results of the GPC analysis revealed a weight average molar mass (Mw) of 364,470 g/mol. Weight average molar mass considers the contribution of each molecule’s weight to the overall average, providing a measure of the overall size of the molecules in the sample. Additionally, a number average molar mass (Mn) of 12,800 g/mol was determined. Number average molar mass considers the total number of molecules in the sample, providing an average size of the individual molecules. These results suggest that the arabinoxylan molecules extracted through alkali extraction have a wide range of molecular weights, with the weight average molar mass being significantly higher than the number average molar mass [33].

### 3.5. Viscosity and pH determinations

A viscosity test is recommended for semi-solid dosage forms in order to investigate the flow properties. The viscosity of the different emulgel formulations was in the range of 10000-30000cps, whereas, the ideal range for the viscosity is 12500–21100 cps. The outcomes of the viscosity studies, as illustrated in Table 1, show that formulation containing 1.5% AX had the value of viscosity within the ideal range. On one hand, a lower concentration (1% AX) was either at a lower borderline or below the ideal range of viscosity and on the other hand, a higher concentration (2% AX) exhibited the value of viscosity either at the upper borderline or above the range of viscosity. By increasing the concentration of AX, the absorption capacity of water decreases due to cross-linking and a complex network of polymers [34].

The pH values of the emulgel formulations, as indicated in Table 1, fell within the range of 5.6 to 6.8. This pH range ensures minimal risk for irritation and is compatible with the skin’s physiology. The natural pH of the skin, typically ranging from 4.5 to 5.5, is vital for maintaining the skin’s barrier function and overall health. Deviations from this optimal pH range can lead to adverse effects such as irritation, dryness, and compromised barrier function. Since emulgel formulations are directly applied to the skin, it is crucial to consider their pH to ensure compatibility and minimize the risk of irritation. By formulating emulgel with a pH close to the skin’s natural pH, we can preserve the integrity and functionality of the skin, maintain microbial balance, and promote optimal skin health. Therefore, maintaining a pH range similar to that of the skin in emulgel formulations is essential for ensuring compatibility, minimizing the risk of irritation, and supporting overall skin health [35].

### 3.6 Stability testing

The emulgel was examined visually for phase separation, pH and content uniformity. Among all formulation, F1, F3 and F4 were found to be incubated under given conditions, whereas all other formulations undergone phase separation. Formulations appear to be creamy with a smooth homogeneous texture and glossy appearance. Table 6 indicates results of stability testing F1, F3 and F4 formulations.

**Table 6:** Outcomes of the stability studies of optimized formulations.

	Formulation											
	F1				F3				F4			
**Days**	**0**	**30**	**60**	**90**	**0**	**30**	**60**	**90**	**0**	**30**	**60**	**90**
**Colour**	x	x	x	x	x	x	x	x	x	x	x	x
**Appearance**	xx	xx	xx	xx	xx	xx	xx	xx	xx	xx	xx	xx
**pH**	5.6± 0.05	5.7 ± 0.03	5.8 ± 0.02	5.0 ± 0.09	6.8 ± 0.05	6.8 ± 0.04	6.5 ± 0.01	6.3 ± 0.06	6.1 ± 0.01	6.0 ± 0.03	5.9 ± 0.02	6.1± 0.01
**Drug content**	106 ± 0.09	105 ± 0.03	105 ± 0.06	102 ± 0.05	117 ± 0.05	115 ± 0.04	116 ± 0.05	115 ± 0.03	85 ± 0.05	84 ± 0.10	84± 0.05	82± 0.03
**Phase separation**	No	No	No	No	No	No	No	No	No	No	No	No

x = Creamy.

xx = Homogeneous.

### 3.7. Spreadability and determination of extrudability

**Fig 3:**
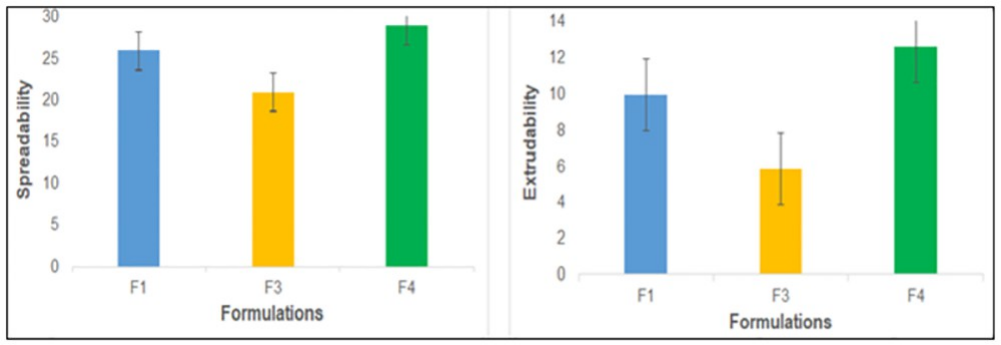
Spreadability and extrudability of the optimized emulgel formulations.

Spreadability is inversely proportional to the viscosity, the less viscous formulation exhibits more spreadability and vice versa [36,37]. The spreadability of the optimized formulations, as given in Fig 3, was found to be in the required range for good spreadability (20-29g.cm/sec). Moreover, it was found that extrudability of emulgel was a function of viscosity and concentration of gelling agent AX in formulations. Extrudability decreased with an increase in the viscosity and concentration of AX in these formulations, 1.5%, 2% and 1% for formulations F1, F3 and F4, as depicted in Fig 3.

### 3.8. Zeta potential, droplet size distribution and PDI

When formulating a stable emulgel system, zeta potential, droplet size distribution, and PDI are the most important factors to consider [38]. The emulgel stability is determined by the amplitude of the zeta potential. The charge on the surface of dispersed globules in an emulgel formulation is measured by the zeta potential. When all particles of emulgel have large positive or negative zeta potential, the repulsion forces between them increase and dispersion stability is achieved. There is no force to keep the particles away from colliding if their zeta potentials are low, resulting in dispersion instability. The ideal range of zeta potential for emulgel stability is +50mV to -50mV.

**Fig 4:**
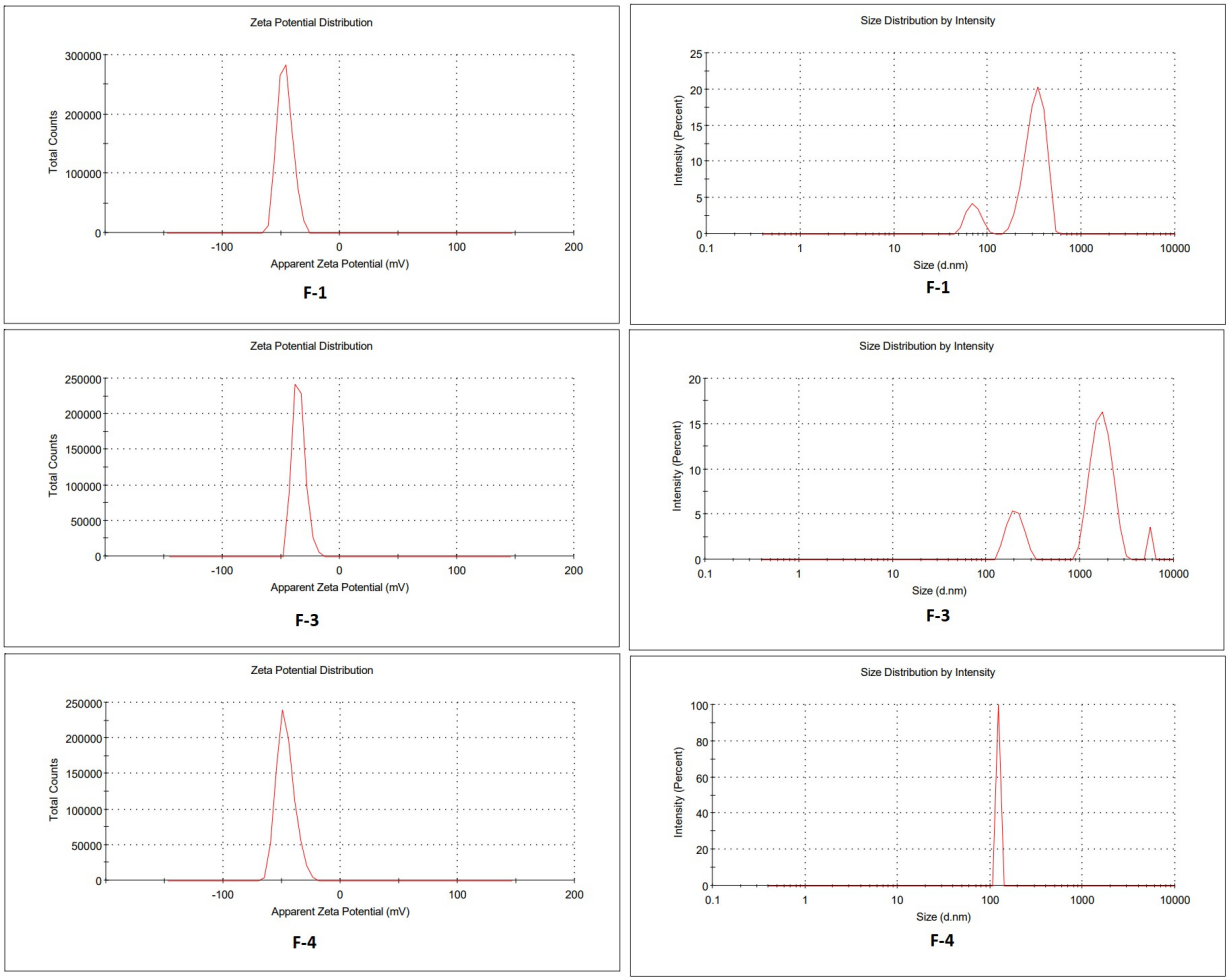
Zeta potential and globule size distribution of Formulation F1, F3, F4.

Fig 4 indicates the zeta potential of formulations F1, F3, and F4 was -46.5, -34.7 and -46.5 respectively. The negative zeta potential is beneficial for enhanced stability of the formulations. Emulgel formulations with either negative or positive zeta potential, i.e., surface charges, have enhanced stability. This is because of the increased electrostatic repulsion between the droplets or globules of the emulsion system and thus it will prevent droplet coalescence. The value of zeta potential lies in the range which confirms the stability of emulgel formulations.

When formulating a stable emulgel system, several factors are crucial to consider, including zeta potential, droplet size distribution, and Polydispersity Index (PDI) [38]. Zeta potential is a measure of the electrostatic charge on the surface of dispersed particles in a colloidal system like emulgel. It significantly influences the stability of the system. In emulgel, negative zeta potential values are generally favorable for enhanced stability. This negative charge arises from the ionization of functional groups present on the particle surface, such as the charged head groups of surfactants or emulsifiers. The negative charge creates electrostatic repulsion between the particles, preventing their close proximity and aggregation. Consequently, the emulgel formulation maintains a dispersed state and exhibits improved stability. Additionally, negative zeta potential can reduce the likelihood of coalescence and phase separation of the oil droplets within the gel matrix, contributing to the overall stability of the emulgel. However, it is important to optimize the zeta potential within an appropriate range as excessively high negative values may introduce other stability challenges.

The optimal range for zeta potential in ensuring emulgel stability is typically between +50mV and -50mV. It is crucial to note that the specific zeta potential value within this range is significant for ensuring the stability of emulgel formulations In Fig 4, the zeta potential values for formulations F1, F3, and F4 were observed to be -46.5, -34.7, and -46.5, respectively. These negative zeta potential values indicate enhanced stability of the formulations. By having zeta potential values within the recommended range, the stability of the emulgel formulations is confirmed.

The particle size of droplets is usually greater than 400 nm, which leads to the opacity of emulgel. The measured size of the particles of F1, F3 and F4 was 872 ± 7.4525 nm, 1168 ± 9.4525 nm and 1828 ± 17.4525 nm. The term PDI represents the system’s polydispersity and its values are 0.0 to 1.0. The PDI for nimesulide emulgel formulations ranged from 0.945,0.975 and 1.00 for F1, F3 and F4, respectively and these values depict the heterogenous dispersion of droplets in the formulation [39].

### 3.9. Photomicrography

**Fig 5:**
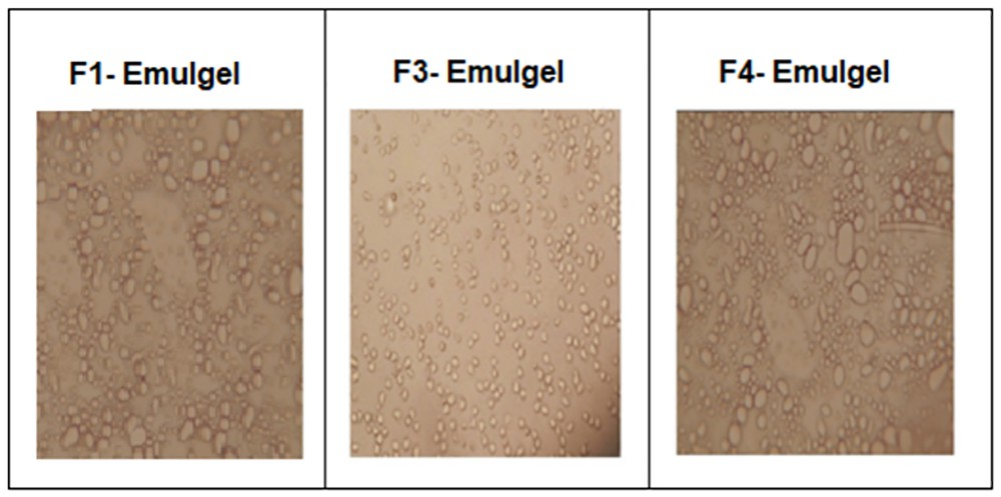
Microscopic view of optimized emulgel formulations F1, F3 and F4.

Fig 5 demonstrates the microscopic view of emulgel formulations F1, F3 and F4. The presence of oil droplets of the emulgel can be seen clearly that act as reservoir for lipophilic drugs and the sustained release of the drug is manifested by the complex gel structure making them ideal carriers for drug delivery.

### 3.10. Ex-vivo permeation study

**Fig 6:**
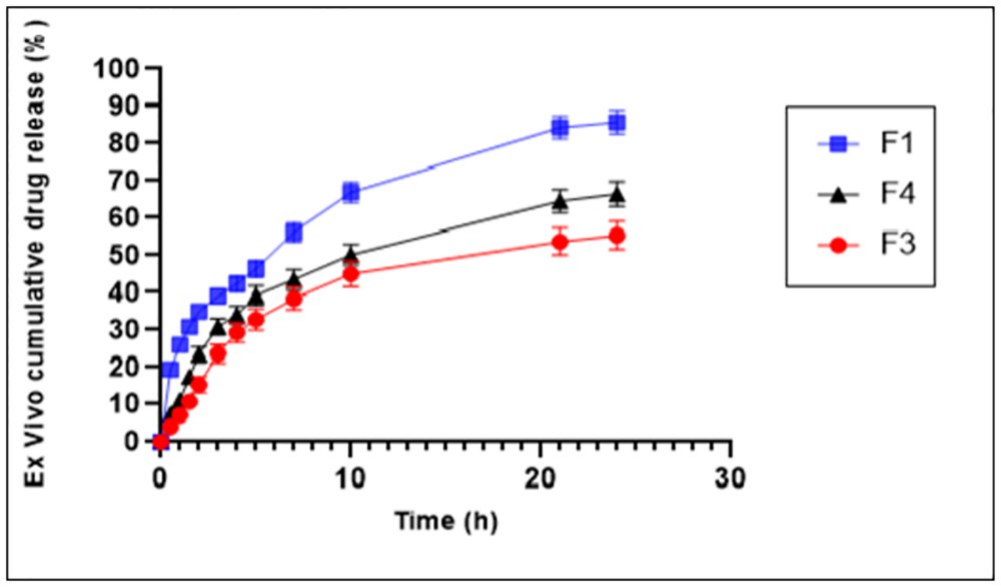
Ex-vivo cumulative % release of nimesulide from formulations F1, F3 and F4.

The release of drug from emulgels depends on multiple factors including gelling agent concentration, spreadability, emulsifying agent, and viscosity. Fig 6 elaborates the ex-vivo permeation study of the model drug, nimesulide. The average release of nimesulide from the formulations F1, F3 and F4 was 86%, 55% and 66%, respectively, within 24 h. The differences in drug permeation can be attributed to the concentration of the gelling agent, specifically the polymer AX, which was approximately 1.5%, 2%, and 1% in F1, F3, and F4, respectively. Formulation F3 displayed incomplete drug permeation, which could be associated with its slightly higher concentration of the polymer AX. The increased polymer concentration might result in the formation of a more complex gel network, hindering the diffusion and release of the drug. This could explain the lower drug permeation observed in F3 compared to F1 and F4 [40]. Another factor influencing drug permeation is the viscosity of the emulgel, which is affected by the swellability of the polymer AX. In our study, the viscosity played a significant role in the overall release of the drug. Higher cross-linking density in F3, resulting from the higher polymer concentration, might lead to decreased elasticity of the polymeric structure [41]. This reduced elasticity could restrict the movement of the drug solution, contributing to the incomplete drug permeation.

Optimized formulation F1 having maximum ex-vivo cumulative drug permeation was therefore selected for FTIR, in-vivo analgesic activity and skin irritation test. Ex-vivo cumulative drug permeation studies provide valuable insights into the release profile of the drug from the formulation. Formulation F1 demonstrated the highest ex-vivo cumulative drug permeation compared to other formulations under investigation. This indicates that F1 has the potential to deliver the drug effectively and efficiently to the target site, which is crucial for achieving the desired therapeutic effect. Furthermore, selecting formulation F1 for additional tests, such as FTIR analysis, in-vivo analgesic activity, and skin irritation testing, provides further insights into its performance and suitability. Additionally, conducting skin irritation tests is crucial to evaluate the formulation’s compatibility with the skin. By assessing the potential for skin irritation, we can ensure that formulation F1 is safe for topical application and minimizes the risk of adverse reactions or irritations.

### 3.11. FTIR analysis

**Fig 7:**
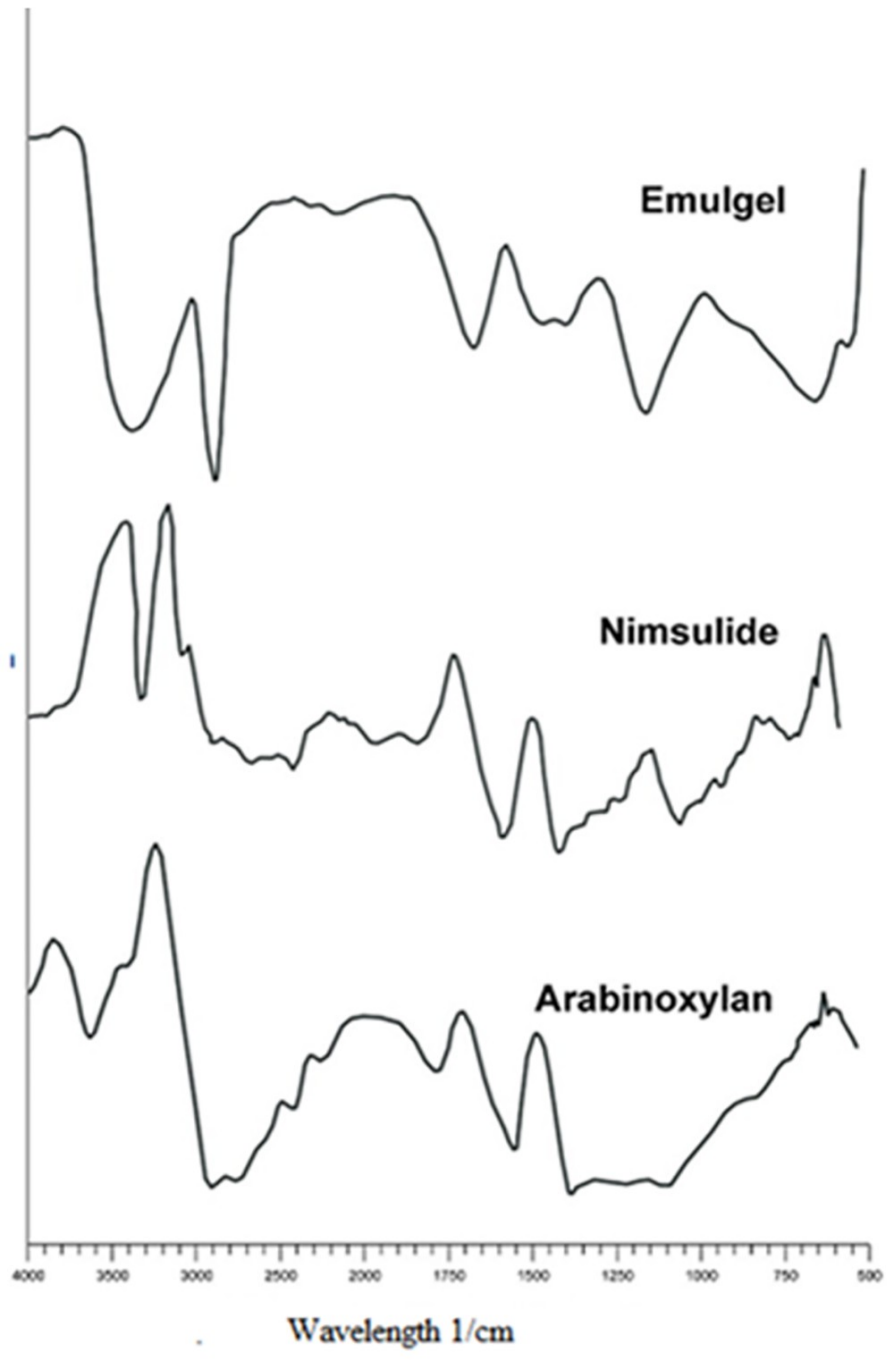
FTIR of AX, pure nimesulide and optimized emulgel formulation F1.

FTIR of AX, pure nimesulide, and optimized emulgel formulation F1 is described in Fig 7. In the FTIR spectrum of AX, the absorption bands at 3595 cm^-1^ indicate OH-stretching, the main band at 2866cm^-1^ is linked with the aliphatic CH-stretching and the bands from 1750–2000 cm^-1^ illustrate the deformation by water absorption. A further look indicates the presence a peak at 1483 cm^-1^ (conforming to the CH_2_ group), 1352 cm^-1^ (CH), 1134 cm^-1^ (arabinosyl side chain) and the peak at 1010 cm^-1^ showing C-C, C-O, and C-O-H group stretching [42]. FTIR spectrum of pure nimesulide provides the fingerprint region from 2500–4000 cm^-1^. The individual strong peak was observed at 3423 cm^-1^ that revealed the N-H stretching (amide group). The peak of the drug at 1490 cm^-1^ confirms the presence of NO_2_ group, while the peak at 1315. 2 cm^-1^ reveals the presence of CH3 group and 1157. 20 cm^-1^ is for S = O group that confirm the drug purity [43]. FTIR of emulgel containing nimesulide clearly confirms the presence of the drug in the emulgel. The sharp peak of nimesulide at 3423 cm^-1^is clear in the spectrum, revealing the presence of amide group of drugs in emulgel.

### 3.12. In-vivo analgesic activity

**Fig 8:**
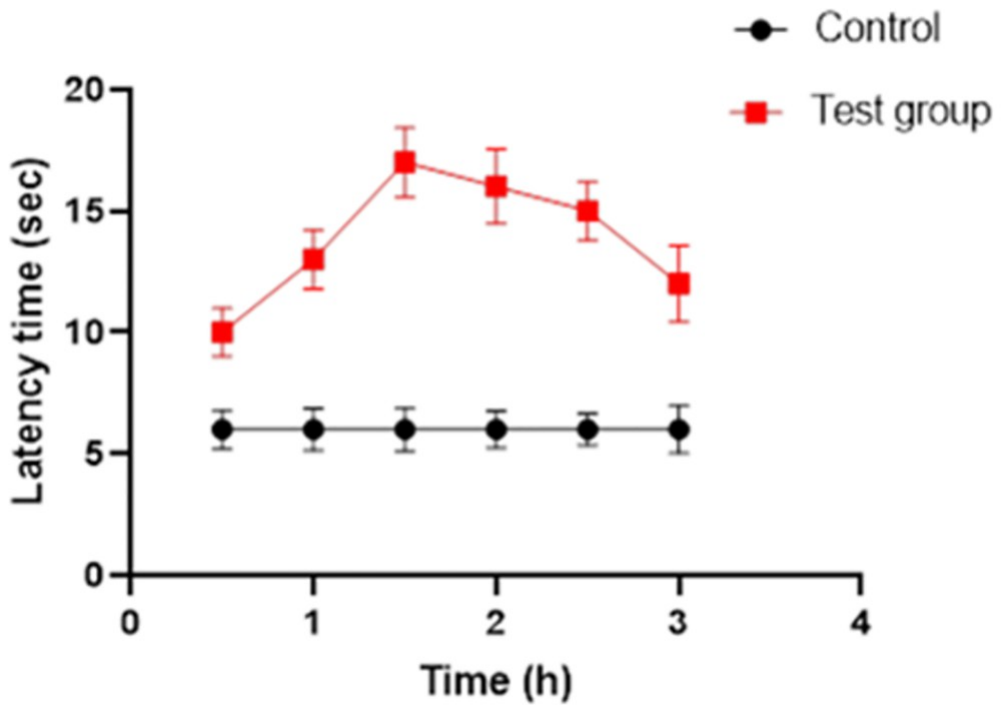
Pain reaction time in test and control group.

Analgesic effect in treated groups of rats and control was evaluated by observing the response (raising or licking paw, jumping) to pain stimulus and outcomes of the study are described in Fig 8. The F1 treated group demonstrated an overall hike in the lapse time compared to control group and therefore, hinting a higher analgesic effect. It was observed that the treated group produced a maximum delay in reaction time (analgesic effect) at 1.5 h that was approximately three folds higher than the control group. The possible reason of better analgesic effect in treated group indicates that drug shows more efficacy in emulgel formulation. PEG was used in nimesulide emulgel which act as a permeation enhancer. After application, integrity of stratum corneum got changed, leading to enhanced percutaneous absorption of developed formulation as compared to marketed product [44]. Statistically, the outcomes of the study, analgesic effect, were found significantly different (*p* < 0.05) for the treated group as compared to control group in the student’s *t*-test.

### 3.13. Skin irritation test

**Fig 9:**
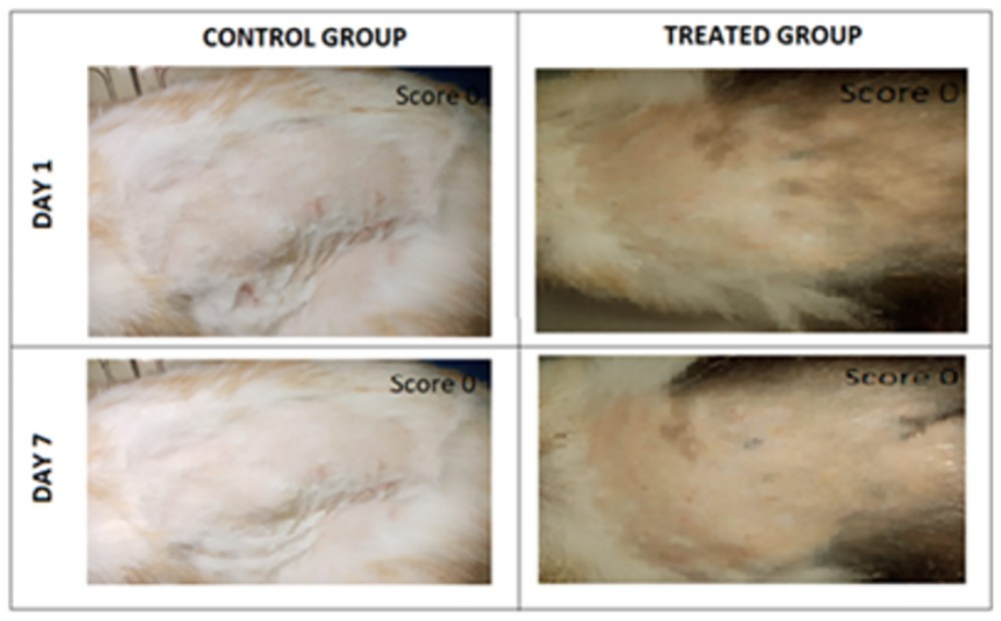
Skin irritation test in control and treated group.

For skin irritation test, procedure for the skin irritation grading was conducted every day extended over a period of one week. As depicted in the Fig 9, the signs of skin irritation such as significant redness or abrupt change in morphology was found absent for both the treatment and control groups. A very mild redness was observed but that is considered a normal skin reaction to the repetitive rub-on of the topical formulation on the same spot. The scoring system from grade 0 to grade 3 was used to estimate skin irritation. In scoring system, score 0 value meant no signs of irritation, score 1 indicated slightly patchy erythema, score 2 patchy erythema and a score of 3 severe erythema with or without edema [45].

The outcomes of the skin irritation via the scoring system are demonstrated in Table 7.

**Table 7:** Evaluation of optimized emulgel for skin irritation in control and treated group.

	Day 1	Day 2	Day 3	Day 4	Day 5	Day 6	Day 7
Control Group	0	0	0	0	0	0	0
Treated Group	0	0	0	0	0	0	0

## 4. Conclusion

This study was designed to evaluate natural biomaterial AX as gelling agent in emulgel formulation.

The findings demonstrated that AX extracted from Plantago ovatago exhibited excellent gelling properties in emulgel formulations. The formulated emulgel, incorporating AX, exhibited desirable characteristics such as homogeneity, spreadability, stability, and gradual drug permeation. The application of response surface methodology aided in optimizing the formulation, and the most promising formulation, in terms of drug permeation, was selected for further investigations. The treated group using the optimized formulation (F1) showed a significant three-fold delay in the onset of analgesic effect compared to the control group. This result clearly indicated the enhanced analgesic activity of the developed formulation. Therefore, it can be concluded that the developed emulgel containing nimesulide demonstrated improved analgesic activity by effectively penetrating the stratum corneum. This formulation holds potential as a carrier for delivering other drugs in future applications. Additionally, the cost-effectiveness of the formulation is achieved through the utilization of readily available and cost-efficient ingredients, making it a viable option for commercial production. These cutting-edge features of our research contribute to advancing the field of topical drug delivery, offering improved efficacy and economic benefits, which can have a significant impact on patient care and treatment outcomes.

## Supporting information

S1 File (RAR)
